# Parental attachment and problematic smartphone use in adolescents: the chain-mediated role

**DOI:** 10.3389/fpsyg.2025.1648291

**Published:** 2025-10-03

**Authors:** Yuan Peng, Wenhua Cui, Ruqian Yang, Hua Wang

**Affiliations:** ^1^Psychology Department at the Teacher School, Xi’an University, Xi’an, China; ^2^Key Laboratory of Children’s Cognitive Science and Education Promotion, Xi’an, China

**Keywords:** parental attachment, problematic smartphone use, peer attachment, online need satisfaction, adolescent

## Abstract

**Aim:**

This study explores how parental attachment influence adolescents’ problematic smartphone use (PSU), examining the chain mediating roles of peer attachment and online need satisfaction in this relationship.

**Methods:**

A chain mediation analysis was conducted using data collected from a sample of 1,208 adolescents who completed questionnaires assessing parent/peer attachment, online need satisfaction, and PSU.

**Results:**

The results showed that (1) parental attachment were negatively associated with PSU; (2) parental attachment influenced PSU through the mediating effect of peer attachment; (3) parental attachment influenced PSU through the mediating effect of online need satisfaction; and (4) peer attachment and online need satisfaction played a chain mediating role in the influence of parental attachment on adolescents PSU.

**Conclusion:**

These findings can help researchers and educators better understand the underlying mechanisms of the relationship between parent and adolescents on PSU and to provide practical and effective operational suggestions for the prevention and intervention of PSU among adolescents.

## Introduction

Problematic smartphone use (PSU) is defined as the manifestation of maladaptive psychological symptoms arising from excessive dependence and compulsive use of the smartphone, leading to addiction-related symptoms characterized by distress or functional damage, as well as non-addiction-related symptoms characterized by escapism (Chang and Ko, 2023). As suggested by previous studies (Bae, 2015; Griffiths, 2017), PSU could be identified by the presence of several core components, including develop dependence and craving for the smartphone, mood modification, loss of control, withdrawal symptoms, conflict, and functional impairment. Adolescence is a high-risk period for PSU, which is associated with various adverse outcomes, including academic procrastination (Parmaksiz, 2022), sleep disorders (Lunsford-Avery et al., 2024), negative emotions (Busch and McCarthy, 2021), and impaired cognitive functioning (Fekih-Romdhane et al., 2022; Loid et al., 2020; Yaakoubi et al., 2024). Consequently, exploring the factors influencing PSU and its underlying mechanisms is crucial for developing strategies to mitigate its prevalence among adolescents.

### Relationship between parental attachment and adolescents’ PSU

Parents play a central role in the social development of children and continue to significantly influence adolescent development. Parental attachment, defined as the enduring emotional bond between parents and their children (Bowlby, 1973), has a profound impact on child and adolescent development. Research has found that adolescents who exhibit secure parental attachment tend to possess effective emotion regulate strategies (Olmeda-Muelas et al., 2024), character strengths (Liu and Wang, 2021), strong self-control, strong self-efficacy, high resilience (Zhou et al., 2023) and a lower tendency to prioritize short-term hedonic gratification (Yee and Shiota, 2015). These positive traits reduce their susceptibility to developing problematic smartphone use (PSU). In contrast, individuals with insecure parental attachment exhibit more fear of missing out (Wang et al., 2024), struggle with the development of social functioning and maintaining healthy relationships in real life. Consequently, they are more likely to engage in compensatory behaviors such as excessive uses smartphones. Previous research has indicated that insecure parental attachment is associated with adolescents’ PSU (Badenes-Ribera et al., 2019; Olmeda-Muelas et al., 2024). A meta- analysis of 167 studies has shown that a negative correlation between attachment security and problematic internet use (Li et al., 2024). The present study aims to further explore the potential psychological mechanisms underlying the relationship between parental attachment and adolescents’ PSU.

### The mediating role of peer attachment

For adolescents, peers become particularly important as long-term companions, offering them emotional support and strength. Some adolescents may be more inclined to communicate and interact with their peers, leading to the development of peer attachment. Peer attachment refers to a deep, stable, and enduring emotional bond formed between individuals and their peers (Bowlby, 1973). The internal working model (Bowlby, 1973) suggests that early attachment patterns that is parental attachment, influence the formation of later attachment relationships, such as peer attachment. Research has shown that adolescents with high-quality parental attachments are more likely to develop expectations and beliefs about stable relationships, exhibit more positive social attitudes, and engage more actively and openly in interactions with peers (Mikulincer and Shaver, 2023). Therefore, high-quality parental attachment relationships facilitate the formation of positive peer attachment relationships.

On the other hand, peer attachment may influence PSU. According to the compensatory internet use theory (Kardefelt-Winther, 2014), individuals with weaker social skills often turn to the internet to compensate for deficiencies in face-to-face interpersonal relationships. Adolescents with poor peer attachment relationships may struggle to establish intimate face-to-face connections, leading them to rely on the internet and smartphone for social interaction and support, thereby increasing their risk of PSU. Research has shown that positive peer relationships serve as important protective factors against adolescent PSU (Huang and Wang, 2022). For instance, a study on high school students found that poor peer attachment was closely associated with internet addiction, which is similar to PSU, with mother-child and father-child attachment directly or indirectly influencing internet addiction through peer attachment. Therefore, the present study proposes that peer attachment may mediate the relationship between parental-child attachment and adolescents’ PSU.

### The mediating role of online need satisfaction

Online need satisfaction refers to individuals using online communication services on the internet or smartphone to fulfill various psychological needs, serving as an important motivational factor for adolescent development (Ryan and Deci, 2020). High satisfaction of psychological needs is significantly associated with physical and mental wellbeing, while low satisfaction can lead to behavioral problems, including addiction (Ryan and Deci, 2020; Van den Berg and Cramer, 2021). According to the self-determination theory, a positive parent-child relationship can fulfill the basic psychological needs of adolescents. Adolescents who experience negative parent-child relationships often lack emotional warmth and effective social support from their parents, which may drive them to use smartphones to seek warmth and support, thereby increasing their online need satisfaction.

The compensatory internet use of need satisfaction theory (Liu et al., 2016) proposes that when adolescents’ psychological needs are not met in real life, they may seek compensation in the online world, which could lead to increased smartphone use. Previous studies have found that online need satisfaction is positively associated with PSU (Błachnio et al., 2023). For example, Steiner and Schneider (2023) found that the higher levels of online need satisfaction correlated with greater online engagement. Additionally, individuals with high communication needs are more likely to use smartphones frequently and are at a higher risk of addiction. The study revealed that adolescents with stronger belongingness needs are more susceptible to smartphone addiction, and positive peer relationships help fulfill these belongingness needs. They also found that adolescents who lack satisfaction in relatedness needs are more likely to repeatedly check their phones for messages and spend excessive time on their phones. Therefore, the present study proposes that online need satisfaction may mediate the relationship between parental-child attachment and adolescents’ PSU.

### Chain mediation of peer attachment and online need satisfaction

**FIGURE 1:**
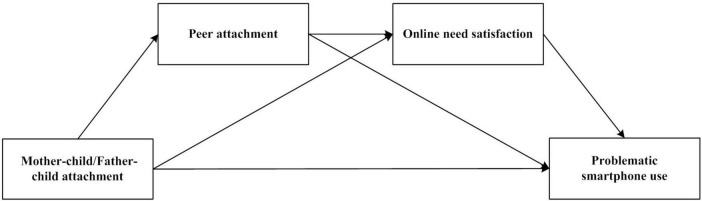
Proposed the chain mediation model.

Furthermore, peer attachment and online need satisfaction are hypothesized to play a chain-mediating role in the relationship between parental attachment and adolescents’ PSU. Specifically, adolescents with lower levels of parental attachment may experience higher peer alienation, which reflects poorer quality of peer relationships. This, in turn, could drive a greater reliance on online platforms to fulfill unmet social needs—such as seeking friendship, support, and social capital through virtual interactions. The compensatory use of social technologies to alleviate real-life relational deficits may enhance motivation for online engagement, thereby increasing the risk of PSU. Thus, within the proposed chain mediation model, peer attachment and online need satisfaction serve as sequential mediators: parental attachment influences peer attachment, which then affects online need satisfaction, and ultimately contributes to PSU. In summary, the present study also examines the chain-mediating effects of peer attachment and online need satisfaction on the relationship between parental attachment and adolescent PSU (see Figure 1 for the proposed model).

Based on the existing theories and research, this study proposes four hypotheses:

Parental attachment is significantly negatively correlated with adolescent PSU;

Peer attachment plays a mediating role in the relationship between father-child/mother-child attachment and adolescent PSU;

Online need satisfaction plays a mediating role in the relationship between father-child/mother-son attachment and adolescent PSU;

Peer attachment and online need satisfaction have a chain-mediated effect between father-child/mother-child attachment and adolescent PSU.

## Methodology

### Participants

Convenience sampling was used to select participants aged 13–18 to take part in the study. With the assistant of schools, parents, and students, 1,300 questionnaires were distributed, and 1,225 were returned. After eliminating invalid questionnaires, 1,208 responses were deemed effective, resulting in an effective recovery rate of 92.9%. Among the participants, 568 were male (47.03%, M*_*age*_* = 16, 41 years, SD = 2.76, range = 13–18 years) and 640 were female (52.97%, M*_*age*_* = 15, 89 years, SD = 2.52, range = 13–18 years). Participants were asked to report their monthly income using a four-point scale (1 = less than ¥3,000, 2 = ¥3,000–¥7,000, 3 = ¥7,000–¥10,000, and 4 = more than ¥10,000) and their education level on a seven-point scale (1 = lower than elementary school, 2 = elementary school, 3 = junior high school, 4 = high school, 5 = college or university, 6 = master’s degree, 7 = doctoral degree). Participants’ socioeconomic status (SES) was calculated using an index consistent with previous studies (Cohen et al., 2006), which included household income (M = 2.05, SD = 1.25), the father’s education level (M = 3.02, SD = 2.11), and mother’s education level (M = 3.14, SD = 2.08).

### Procedure

The present study was approved by the research ethics committee of the authors’ institute. Informed consent forms were distributed to junior school and junior high school students, inviting them to participate in the study. Consent was obtained from school administrators, parents, and adolescents. The adolescents were informed of their right to refuse participation or withdraw from the study at any time without any negative consequences. They were also provided with details about the nature of the study and assured that their responses would remain confidential and be used exclusively for research purposes. The survey took approximately 20 min to complete. The participants completed the questionnaires in a designated experiment room and received a gift for their participation.

### Measures

#### Problematic smartphone use

The modified problematic smartphone use scale, as developed by Su et al. (2014), was utilized. The scale contains 22 questions across six dimensions, including social pacification, negative affect, withdrawal behavior, salient behavior, app use, and app update. Participants rated each item on a five-point scale ranging from 1 (strongly disagree) to 5 (strongly agree), with a higher total score indicating a greater degree of problematic smartphone use. The internal consistency of the scale in this study was measured using the Cronbach alpha coefficient, which was found to be 0.83.

#### Parent/peer attachment

To assess participants’ cognitive perception of and feelings toward their parents and friends, this study utilized a simplified version of the inventory of parental and peer attachment (IPPA-R) (Raja et al., 1992). The IPPA-R consisted of 12 items that measured attachment quality for each parent and peers along the dimensions of trust, alienation, and communication. Participants rated each item on a five-point scale ranging from 1 (strongly disagree) to 5 (strongly agree). Total scores were calculated by summing all items (with some reverse-scored) for each subscale, and a higher score indicated stronger attachment security with parents (maternal and paternal combined) or peers. The Cronbach’s α coefficients were 0.85 for peer attachment, 0.82 for father-child attachment, and 0.74 for mother-child attachment, indicating good internal consistency.

#### Online need satisfaction

The psychological needs online satisfaction scale, as revised by Shen et al. (2013), was utilized. The revised scale includes three dimensions, which are autonomy, competence, and belongingness, each comprising of four questions. For instance, questions such as “I can choose what I like when I am online,” “I feel that my online performance is good,” and “I feel that people know me well when I am online” are used to measure these dimensions. Participants rated each item on a five-point scale ranging from 1 (strongly disagree) to 7 (strongly agree), with “strongly agree” being scored as seven, and a higher total score indicates a greater satisfaction with online psychological needs. The internal consistency of the scale measured through Cronbach’s alpha coefficient in this study was 0.85.

### Data analysis

SPSS 26.0 was used in this study for reliability analysis, descriptive statistics and correlation analysis of the four variables, and Process plug-in V4.1 (Hayes, 2022) was used for the mediation effect test.

## Results

### Common method bias test

The common method bias test using Harman’s one-way test analyzed a total of 11 common factors greater than 1, and the first factor explained 19.70% of the variance, which did not reach the critical criterion of 40%. Therefore, there is no serious common method bias in the variables of this study.

### Correlation analysis among variables

The results of the correlation analysis showed that PSU was significantly positively related to online need satisfaction (*r* = 0.47), and significantly negatively related to mother-child (*r* = −0.17), father-child (*r* = −0.22), and peer attachment (*r* = −0.11) (See Table 1).

**TABLE 1:** Descriptive statistics and correlations among variables.

	1	2	3	4	5	6	7
1. Sex	1						
2. SES	0.02	1					
3. Mother-child attachment	0.08*	−0.02	1				
4. Father-child attachment	−0.01	0.001	0.56**	1			
5. Peer attachment	−0.08*	0.002	0.40**	0.38**	1		
6. Online need satisfaction	0.01	0.01	−0.06	−0.09*	0.07*	1	
7. PSU	0.05	−0.06	−0.17*	−0.22*	−0.11*	0.47**	1
M	–	–	42.74	35.00	43.2	53.73	67.68
SD	–	–	7.08	7.60	6.87	14.03	16.43

*p < 0.05, **p < 0.01, ***p < 0.001. PSU, problematic smartphone use.

### Analysis of the chain-mediated effects between mother-child attachment and PSU

In the preliminary correlation analysis, all variables except for the non-significant relationship between mother-child attachment and online need satisfaction were found to be significantly correlated with each other. Furthermore, the correlation between the two mediating variables-peer attachment and online need satisfaction- was significant. Therefore, a chain mediation could be conducted (Wen and Ye, 2014).

**FIGURE 2:**
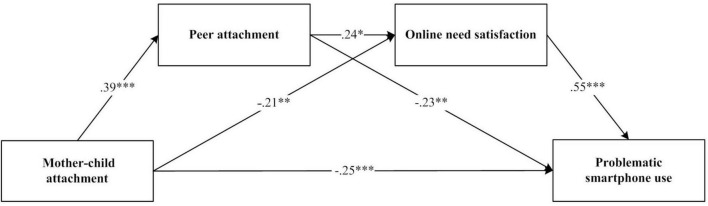
Chain mediation model between mother-child attachment and problematic smartphone use. *p < 0.05, **p < 0.01, ***p < 0.001.

After controlling for sex and grade, the data were analyzed using the Process plug-in v 4.1. Model 6 was employed, introducing two mediating variables: peer attachment and online need satisfaction. Using the bias-corrected percentile bootstrap method, 5,000 samples were extracted to calculate the 95% confidence intervals (Fang et al., 2012). As shown in Figure 2, a multiple chain mediation model was construct. The results of the mediating effect are presented in Tables 2, 3.

**TABLE 2:** The mediating effect between mother-child attachment and PSU.

Predictors	Outcome: peer attachment		Outcome: online need satisfaction		Outcome: PSU	
	*B*	*t*	*B*	*t*	*B*	*t*
Mother-child attachment	0.39	13.31***	−0.21	−2.96**	−0.25	−3.36**
Peer attachment			0.24	3.20**	−0.23	−3.05**
Online need satisfaction					0.55	16.12***
*R* ^2^	0.16		0.02		0.25	
*F*	177.14		6.77		99.33	

*p < 0.05, **p < 0.01, ***p < 0.001. PSU, problematic smartphone use.

**TABLE 3:** Mediated effects and confidence intervals.

Intermediary path	Efficiency value	Bootstrap SE	Proportion of total effect	95% confidence interval	
Mother-child attachment → peer attachment → PSU	−0.09	0.03	22.56%	−0.16	−0.03
Mother-child attachment → online need satisfaction → PSU	−0.12	0.04	28.11%	−0.20	−0.04
Mother-child attachment → peer attachment → online need satisfaction → PSU	0.05	0.02	12.59%	0.02	0.09
Total indirect effect value	−0.16	0.05	38.73%	−0.25	−0.06

### Analysis of the chain-mediated effects between father-child attachment and adolescents’ PSU

**FIGURE 3:**
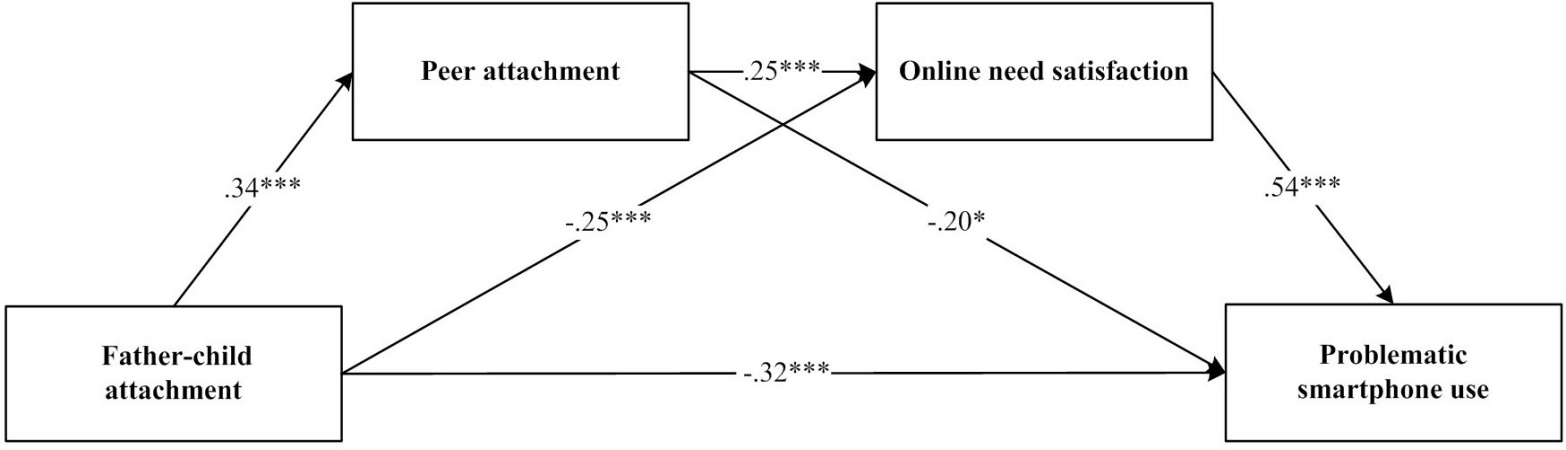
Chain mediation model between father-child attachment and problematic smartphone use. *p < 0.05, ***p < 0.001.

From the results of the correlation analysis, it is evident that four variables: father-child attachment, peer attachment, online need satisfaction and PSU significantly correlated with each other. This finding satisfies the prerequisite for conducting a multiple chain mediation analysis. Therefore, after controlling for sex and grade, the data were analyzed using Process v4.1. Model 6 was applied, introducing two mediating variables: peer attachment and online need satisfaction. The bias-corrected percentile Bootstrap method was used to extract 5,000 samples and calculate the 95% confidence intervals (Fang et al., 2012). As shown in Figure 3, a multiple chain mediation model was construct. The results of the mediating effects are presented in Tables 4, 5.

**TABLE 4:** The mediating effect test between father-child attachment and PSU.

Predictors	Outcome: peer attachment		Outcome: online psychological needs satisfaction		Outcome: PSU	
	*B*	*t*	*B*	*t*	*B*	*t*
Father-child attachment	0.34	12.44***	−0.25	−3.84***	−0.32	−4.75***
Peer attachment			0.25	3.50***	−0.20	−2.64**
Online need satisfaction					0.54	15.89***
*R* ^2^	0.15		0.02		0.26	
*F*	154.81		9.80		104.27	

*p < 0.05, **p < 0.01, ***p < 0.001. PSU, problematic smartphone use.

**TABLE 5:** Mediated effects and confidence intervals.

Intermediary path	Efficiency value	Bootstrap SE	Proportion of total effect	95% confidence interval	
Father-child attachment → peer attachment → PSU	−0.07	0.03	14.26%	−0.13	−0.02
Father-child attachment → online need satisfaction → PSU	−0.14	0.04	28.48%	−0.21	−0.07
Parent-child attachment → peer attachment → online need satisfaction → PSU	0.05	0.01	9.91%	0.02	0.08
Total indirect effect value	−0.16	0.04	32.83%	−0.25	−0.07

*p < 0.05, **p < 0.01, ***p < 0.001. PSU, problematic smartphone use.

## Discussion

### Relationship between parental attachment and PSU in adolescents

The present study found a significant negative correlation between both mother-child attachment and father-child attachment and adolescents’ PSU, confirming Hypothesis 1. These findings are consistent with previous research, demonstrating that strong parental attachment significant impacts PSU. The mother’s role is particularly crucial in adolescent development. Trust and communication between mothers and adolescents can foster a sense of security and care, promoting healthy attachment styles. High-quality mother-child attachment can also mitigate internalizing problems such as loneliness and anxiety (Zhao and Wang, 2023). Conversely, poor mother-child attachment may lead to pessimism toward the world and a cold attitude in interpersonal interactions. Such long-term negative emotional and behavioral patterns could adversely affect adolescents’ future relationships and social adaptation. The quality of the mother-child may serve as an indicator of PSU, with poor relationships potentially driving adolescents to escape through excessive phone use. Similarly, father-child attachment also plays a significant role in adolescent PSU. Fathers contribute substantially to healthy childhood and adolescence development. If fathers are neglectful, poor father-child attachment may lead adolescents to rely excessively on their phones to fulfill unmet psychological needs, thereby increasing the risk of PSU. The finding suggests that as fathers become more involved in child-rearing and take greater responsibility for the education and discipline of their children, their roles as caregivers grow increasingly similar to those of mothers (Fagan et al., 2014). Overall, the present study is consistent with previous research (Açikel and Çetin, 2024; Kocyigit et al., 2021), suggesting that parents play crucial roles in adolescents’ smartphone use.

### The mediating role of peer attachment

The study revealed that both father-child and mother-child attachment have direct and indirect effects on adolescent PSU, with peer attachment partially mediating this relationship. This suggests that the influence of parental attachment and PSU can be partially explained by the role of peer attachment. These results are consistent with previous research (El-Asam and Ameen, 2023) and support Hypothesis 2, indicating that peer attachment acts as a significant mediating mechanism between parental attachment and adolescent PSU.

Adolescence is a critical developmental stage during which individuals gradually gain independence from their parents, and emotional bonds with peers become increasingly significant. Consequently, the primary focus of attachment shifts from parents to peers. According to the attachment theory (Bowlby, 1973), parental attachment forms the most fundamental relational bond for children and can influence the quality of peer relationships. Positive emotional coping styles within parent-child relationships can foster healthy interpersonal dynamics, which in turn shape adolescent interactions with their peers. Previous research has shown a significant positive relationship between parental attachment and peer attachment, suggesting that higher- quality parent-child relationships are associated with closer peer connections. The transition to school represents a pivotal period for adolescent independence, and secure parent-child and peer attachment can facilitate smoother navigation of this stage, promoting self-esteem, prosocial behavior and reducing maladjustment.

Moreover, the finding align with the compensatory internet use theory (Kardefelt-Winther, 2014), which posits that individuals with weaker real-life social skills often compensate for interpersonal deficiencies through online interactions. Adolescents with poor parental attachment are more likely to experience weaker peer attachment, making it challenging for them to establish meaningful face-to-face relationships. As a result, they may turn to online social interactions and seek support through digital devices, increasing their susceptibility to smartphone dependence. These results underscore the importance of fostering positive attachment to support healthy adolescent development and mitigate the risk of PSU.

### The mediating role of online need satisfaction

The findings indicate that online need satisfaction partially mediates the relationship between both father-child and mother-child attachment and adolescents’ PSU, partially providing support for Hypothesis 3. This suggests that parental attachment not only directly affects adolescent PSU but also indirectly influences it through online need satisfaction (Zhou and Yu, 2022). Adolescents who experience poor parent-child relationships may lack adequate social support and emotional warmth from their fathers, leading to reduced psychological needs satisfaction. As a result, when these needs remain unmet in the real world, adolescents may engage in maladaptive behavior, such as excessive smartphone use, as a compensatory mechanism to escape real-world pressure and fulfill their sense of emptiness and alienation through virtual spaces. Simultaneously, they may seek emotional warmth and support on line. Thus, poor parental attachment can drive adolescents to seek online need satisfaction, thereby increasing their risk of PSU.

### Chain mediation of peer attachment and online need satisfaction

The present study provides evidence for the chain-mediated effect of peer attachment and online need satisfaction in the relationship between mother-child/father-child attachment and adolescent PSU, confirming Hypothesis 4. These findings suggest that the combination of peer attachment and online need satisfaction serves as a strong predictor of PSU.

On one hand, the study revealed a significant influence of peer attachment on online need satisfaction. During adolescence, individuals gradually become more independent from their parents, and peers play a critical role in their psychological and behavioral development. Positive peer relationships not only facilitate communication but also help adolescents cope with various interpersonal challenges. In the online context, individuals engage in various forms of interaction, such as online chatting and gaming (Varga and Charzyńska, 2022). Adolescents with strong peer relationships are more likely to share their emotions and experience with peers, both online and offline (Khalis and Mikami, 2018), which contributes to the satisfaction their psychological needs.

On the other hand, consistent with previous studies, the study showed a positive relationship between online need satisfaction and PSU. Liu et al. (2023) found that adolescents often compensate for unmet competence needs by sharing positive information and receiving feedback from friends on social networking sites. Additionally, online games can provide adolescents with a sense of competence after completing challenging tasks (Przybylski and Weinstein, 2019). When adolescents experience satisfaction through smartphone use, they are more likely to continue seeking online needs fulfillment through their devices. This cycle of online need satisfaction and smartphone use can contribute to the development of PSU.

In summary, peer attachment and online need satisfaction play a chain-mediated role in the relationship between mother-child/father-child attachment and adolescent PSU. These findings highlight the interconnected pathways through which attachment relationships and online behaviors influence adolescent smartphone use.

### Implications

The model proposed and evaluated in the study demonstrated that parental attachment is negatively associated with PSU among adolescents. Specific strategies for strengthening parent-child attachment, such as establishing regular positive routines and improving emotional communication, could be offered to help mitigate the risk of PSU. Furthermore, peer attachment and online need satisfaction were found to have independent and multiple mediating effects on the relationship between parental attachment and PSU. These finding offer valuable insights for the development of prevention and intervention strategies in the digital age. First, the establishment of a secure attachment base through parent-child relationships equips adolescents with the necessary competencies to maintain healthy social interactions based on affection. Adolescents with high-quality parental attachment are more likely to be accepted by their peer groups and are better able to establish and sustain positive, high-quality relationships.

Second, the results highlight the importance of enhancing peer attachment to reduce the risk of PSU among adolescents. Educators could employ various intervention strategies, such as cognitive behavioral play therapy, to improve peer attachment levels. Strengthening these social bonds may serve as a protective factor against excessive smartphone use.

Third, the study revealed that online need satisfaction has a complex relationship with PSU. While smartphones can fulfill certain psychological needs, excessive reliance on them-particularly in the absence of a secure attachment base through parent-child relationships- may increase the risk of PSU. The findings suggest that enhancing need satisfaction in real-life contexts, such as through supportive teacher-student interactions, can effectively mitigate this risk. For example, when teachers recognize students’ negative emotions and offer guidance to help them cope with difficult situations, students achieve greater need satisfaction, which in turn decreases their reliance on smartphones for emotional fulfillment (Filippello et al., 2019, Hao et al., 2023).

### Limitations

However, several limitations to this study should be acknowledged. First, although a substantial amount of data was collected, the sample size and diversity were still limited, which may affect the generalizability of the findings. Secondly, as a cross-sectional study, it does not establish causal relationships among variables that the direction of causality could potentially be reversed and is susceptible to generational and age-related influences. Future research should consider conducting larger, more diverse, and longitudinal studies to further validate these findings and explore the causal dynamics between the variables.

## Conclusion

In conclusion, the present study revealed significant negative associations between both mother-child and father-child attachments and adolescents’ PSU. Furthermore, peer attachment and online need satisfaction partially mediated the relationship between father-child attachments and PSU, while peer attachment and online need satisfaction played a chain-mediation role between mother-child or father-child attachment relationships and adolescents’ PSU. Such knowledge can inform the design of effective prevention and intervention programs to improve the parental or peer attachment on adolescent smartphone use.

## Data Availability

The raw data supporting the conclusions of this article will be made available by the authors, without undue reservation.
